# Exceptionally rapid response to pembrolizumab in a SMARCA4‐deficient thoracic sarcoma overexpressing PD‐L1: A case report

**DOI:** 10.1111/1759-7714.13215

**Published:** 2019-10-16

**Authors:** Kohichi Takada, Shintaro Sugita, Kazuyuki Murase, Tomoki Kikuchi, Ginji Oomori, Ryo Ito, Naotaka Hayasaka, Koji Miyanishi, Satoshi Iyama, Hiroshi Ikeda, Masayoshi Kobune, Makoto Emori, Junji Kato, Tadashi Hasegawa

**Affiliations:** ^1^ Department of Medical Oncology Sapporo Medical University School of Medicine Sapporo Japan; ^2^ Department of Surgical Pathology Sapporo Medical University School of Medicine Sapporo Japan; ^3^ Department of Surgical Pathology Asahikawa Red‐cross Hospital Asahikawa Japan; ^4^ Department of Hematology Sapporo Medical University School of Medicine Sapporo Japan; ^5^ Department of Orthopedic Surgery Sapporo Medical University School of Medicine Sapporo Japan

**Keywords:** PD‐L1, pembrolizumab, SMARCA4‐deficient thoracic sarcoma

## Abstract

SMARCA4‐deficient thoracic sarcoma (SMARCA4‐DTS) is a new clinical entity characterized by SMARCA4 inactivation and has a dismal prognosis because of rapid growth. Effective treatments for SMARCA4‐DTS have not yet been developed. Most recently, anti‐programmed cell death 1 receptor (PD‐1) blockade has been effective for SMARCA4‐deficient lung cancer and malignant rhabdoid tumor‐like tumors. Here, we describe a patient with SMARCA4‐DTC who experienced a marked response to the administration of pembrolizumab. A 70‐year‐old female was referred to our department for treatment of SMARCA4‐DTC. Positron emission tomography‐computed tomography had revealed a left mediastinal tumor, peritoneal dissemination and multiple cutaneous metastases at diagnosis. Immunohistochemical analyses revealed 60% of tumor cells expressed programmed cell death ligand 1 (PD‐L1). The patient was given pembrolizumab as first‐line treatment. Pembrolizumab suppressed tumor growth dramatically, with only one dose leading to a partial response. Our case suggests the immunohistochemical analysis of PD‐L1 expression be undertaken for patients with SMARCA4‐DTS and that pembrolizumab treatment may be a promising strategy for PD‐L1‐positive SMARCA4‐DTS.

## Key points

### Significant findings of the study

A patient with PD‐L1‐overexpressing SMARCA4‐DTS was successfully treated with pembrolizumab.

### What this study adds

Examining tumor PD‐L1 expression in patients with SMARCA4‐DTS increases therapeutic options.

## Introduction

SMARCA4‐deficient thoracic sarcoma (SMARCA4‐DTS) has been established as a novel category of malignancy, distinct from lung cancer but related to a SMARCB1‐inactivated malignant rhabdoid tumor (MRT) and SMARCA4‐mutated small‐cell carcinoma of the ovary, hypercalcemic type (SCCOHT).^1^, ^2^, ^3^ SMARCA4‐DTS is characterized by lethal and aggressive thoracic tumors induced by the loss of SMARCA4, also termed Brahma‐related gene 1 (BRG1), which is a member of the polybromo‐ and BRG1‐associated factor (PBAF) form of switch‐sucrose nonfermentable chromatin remodeling complexes.^4^

The prognosis for SMARCA4‐DTS cases is poor, with median overall survival around six months because treatment strategies for this disease have not been established.^1^, ^5^

Accumulating evidence has revealed that the clinical benefit of antiprogrammed cell death 1 receptor (PD‐1) blockade was associated with the loss of function of PBAF complexes in a subset of malignancies.^6^, ^7^ Most recently, it has been reported that anti‐PD‐1 blockade was effective for patients with SMARCA4‐deficient tumors.^8^, ^9^ However, the mechanisms of antitumor activities that induced anti‐PD‐1 blockade have not been fully elucidated. In fact, two cases with SMARCA4‐DTS successfully treated by anti‐PD‐1 blockade did not express programmed cell death ligand 1 (PD‐L1) in their tumors.

In this report, we describe a rapid and major response to pembrolizumab in a case of SAMRCA4‐DTS that overexpressed PD‐L1.

## Case report

A 69‐year‐old female presented to a previous hospital with an initial diagnosis of malignant lymphoma by means of biopsy from a right breast tumor. However, clinical signs and pathological findings did not support the diagnosis of malignant lymphoma. Therefore, a second biopsy of another cutaneous tumor in the lower anterior abdomen was carried out. Finally, the patient was diagnosed with SMARCA4‐DTS and referred to our department.

**Figure 1:**
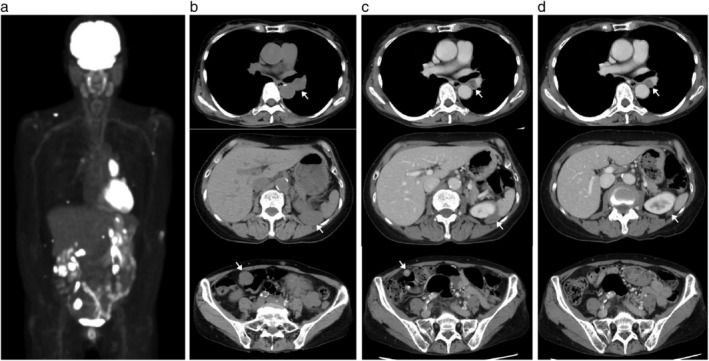
PET‐CT and follow‐up CT images. Arrows indicate tumors. (**a**) Pretreatment positron emission tomography (PET)‐computed tomography (CT) scan of the patient. (**b**) Pretreatment CT scan. (**c**) CT scan after one dose of pembrolizumab. (**d**) CT scan after eight doses of pembrolizumab.

**Figure 2:**
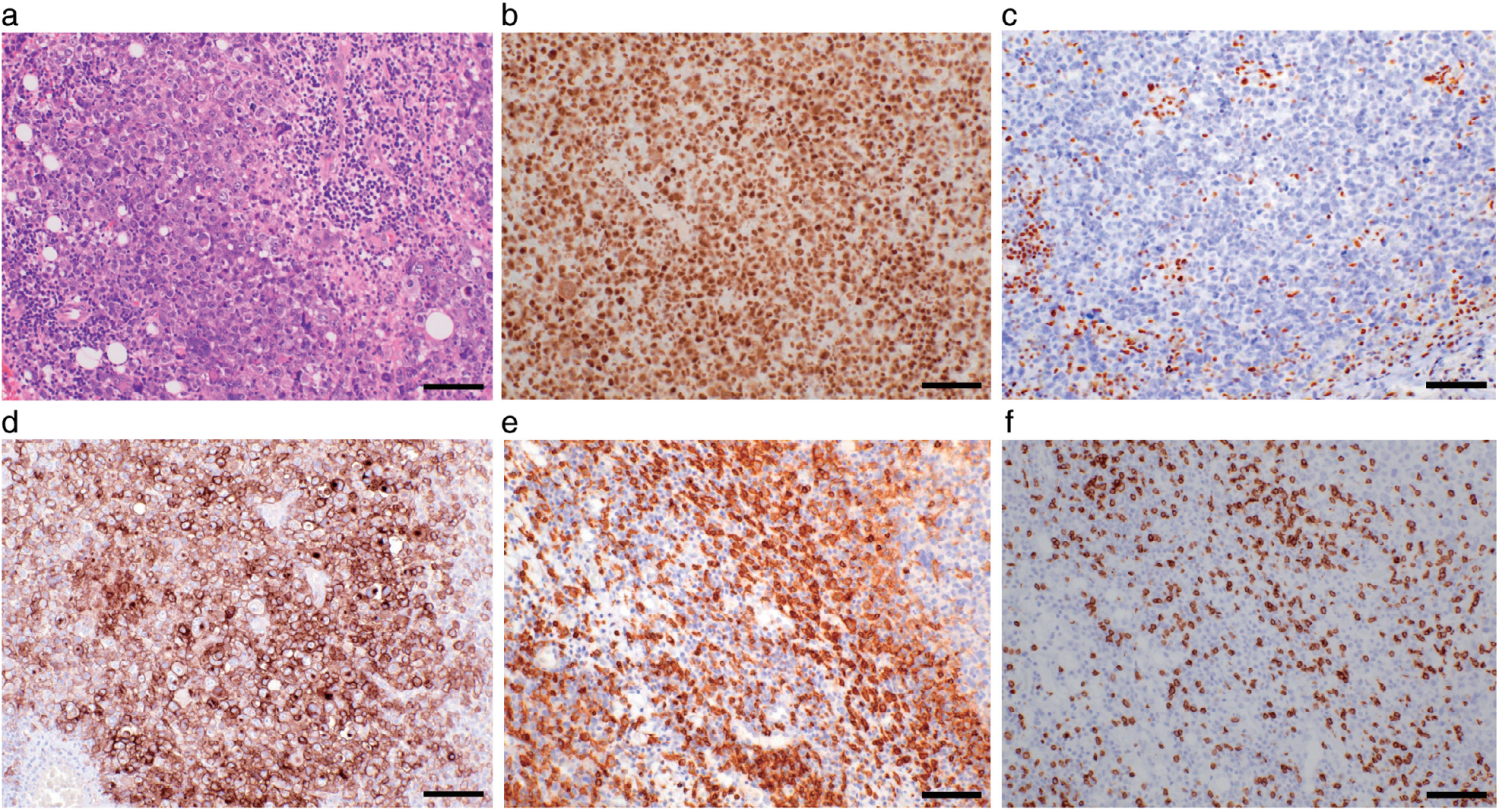
Microscopic findings in a resected tumor. Scale bars 100 μm. (**a**) Hematoxylin and eosin (H&E) staining. (**b**) SMARCA2 (INI1) staining. (**c**) SMARCA4 (BRG1) staining. (**d**) Programmed cell death ligand 1 (PD‐L1) staining. (**e**) CD4 staining. (**f**) CD8 staining.

The patient's main complaints were a palpable multiple cutaneous mass, lower abdominal pain and bloody sputum. A positron emission tomography (PET)‐computed tomography (CT) scan revealed a left mediastinal tumor, peritoneal and retroperitoneal dissemination and multiple cutaneous metastases (Fig 1a,b). Histologically, the tumor consisted of a sheet‐like proliferation of polyhedral tumor cells with an eosinophilic cytoplasm, and enlarged round nuclei with vesicular nuclear chromatin and conspicuous nucleoli (Fig 2a). The resected tumor showed massive foci of necrosis. Small‐sized lymphocytes were intermingled with tumor cells (Fig 2a). On immunohistochemistry, the tumor cells were positive for SMARCA2 (INI1; Fig 2b) and negative for SMARCA4 (BRG1; Fig 2c). The tumor cells also showed high PD‐L1 expression (>60%; Fig 2d). The expression of DNA mismatch repair proteins, such as MLH1, MSH2, MSH6, and PMS2, was retained. Background lymphocytes included both CD4‐positive (Fig 2e) and CD8‐positive T‐lymphocytes (Fig 2f). Microsatellite instability status was stable tested by PCR (Falco Biosystems, Kyoto, Japan). We treated the patient with pembrolizumab as a first‐line treatment because of high PD‐L1 expression in tumor cells. After one pembrolizumab infusion, the patient's abdominal pain and bloody sputum were resolved, suggesting a possible clinical benefit. Strikingly, a CT scan demonstrated a partial response (PR; Fig 1c). A CT scan after eight cycles of pembrolizumab demonstrated a sustained durable PR response with no adverse events (Fig 1d).

Written informed consent was obtained from the patient for the publication of all clinical data and images.

## Discussion

We describe the successful treatment of a patient with PD‐L1 overexpressing SARCA4‐DTS by pembrolizumab. Of note, two previous reports showed that anti‐PD‐1 blockade was effective for patients with SMARCA4‐deficient tumors that, in contrast to our present case, did not express PD‐L1.^8^, ^9^ This is the first case to show overexpressed PD‐L1 in tumors in a patient with SMARCA4‐DTS. The majority of cases with SCCOHT, which is similar to SMARCA4‐DTS, exhibited PD‐L1 expression and massive T‐cell infiltration around tumors that suggested SCCOHT to be an immunogenic tumor.^3^ In fact, four cases of SCCOHT achieved major responses to anti‐PD‐1 blockade. According to the immune phenotypes of SCCOHT, it may be that SMARCA4 defects induce PD‐L1 expression in tumors. However, patients with SMARCA4‐DTS did not express PD‐L1 and show infiltration of T‐cells in tumors, unlike in SCCOHT and our case.^1^, ^8^ The relationship between SMARCA4 deficiencies and immunogenicity needs to be examined further.

The mechanisms that determine sensitivity to anti‐PD‐1 blockade remain to be fully elucidated. Growing evidence suggests such mechanisms include the high expression of PD‐L1, DNA mismatch‐repair deficiency, a high tumor mutational burden, an interferon (IFN)‐γ signature and the presence of CD8+ T cells.^10^, ^11^, ^12^, ^13^, ^14^ In our case, plausible mechanisms for the dramatic response to pembrolizumab may include PD‐L1 overexpressed in tumors and the dysfunction of PBAF associated with the loss of SMACA4. The loss of PBAF function may induce the upregulation of IFN‐γ‐responsive genes and secretion of T cell chemoattractants to recruit effector T cells to tumors.^6^ Therefore, dysfunction of such chromatin remodeling complexes can lead to the increased efficacy of an anti‐PD‐1 blockade against malignant tumors.

Our case suggests that anti‐PD‐1 blockade may be effective for PD‐L1 expressing SMARCA4‐DTC. We should therefore determine the expression of PD‐L1 in SMARCA4‐DTC to consider all treatment options.

## Disclosure

All authors report no conflicts of interest.

## References

[1] Le Loarer F , Watson S , Pierron G *et al* SMARCA4 inactivation defines a group of undifferentiated thoracic malignancies transcriptionally related to BAF‐deficient sarcomas. Nat Genet 2015; 47: 1200–5. 26343384 10.1038/ng.3399

[2] Versteege I , Sévenet N , Lange J *et al* Truncating mutations of hSNF5/INI1 in aggressive paediatric cancer. Nature 1998; 394: 203–6. 9671307 10.1038/28212

[3] Jelinic P , Ricca J , Van Oudenhove E *et al* Immune‐active microenvironment in small cell carcinoma of the ovary, hypercalcemic type: Rationale for immune checkpoint blockade. J Natl Cancer Inst 2018; 110: 787–90. 29365144 10.1093/jnci/djx277 PMC6037122

[4] Kadoch C , Crabtree GR , Mammalian SWI . SNF chromatin remodeling complexes and cancer: Mechanistic insights gained from human genomics. Sci Adv 2015; 1: e1500447. 26601204 10.1126/sciadv.1500447 PMC4640607

[5] Perret R , Chalabreysse L , Watson S *et al* SMARCA4‐deficient thoracic sarcomas: Clinicopathologic study of 30 cases with an emphasis on their nosology and differential diagnoses. Am J Surg Pathol 2019; 43: 455–65. 30451731 10.1097/PAS.0000000000001188

[6] Pan D , Kobayashi A , Jiang P *et al* A major chromatin regulator determines resistance of tumor cells to T cell‐mediated killing. Science 2018; 359: 770–5. 29301958 10.1126/science.aao1710 PMC5953516

[7] Miao D , Margolis CA , Gao W *et al* Genomic correlates of response to immune checkpoint therapies in clear cell renal cell carcinoma. Science 2018; 359: 801–6. 29301960 10.1126/science.aan5951 PMC6035749

[8] Henon C , Blay JY , Massard C *et al* Long lasting major response to pembrolizumab in a thoracic malignant rhabdoid‐like SMARCA4‐deficient tumor. Ann Oncol 2019; 30: 1401–1403. 10.1093/annonc/mdz160. 31114851

[9] Naito T , Umemura S , Nakamura H *et al* Successful treatment with nivolumab for SMARCA4‐deficient non‐small cell lung carcinoma with a high tumor mutation burden: A case report. Thorac Cancer 2019; 10: 1285–8. 30972962 10.1111/1759-7714.13070 PMC6501032

[10] Reck M , Rodríguez‐Abreu D , Robinson AG *et al* Pembrolizumab versus chemotherapy for PD‐L1‐positive non‐small‐cell lung cancer. N Engl J Med 2016; 375: 1823–33. 27718847 10.1056/NEJMoa1606774

[11] Overman MJ , McDermott R , Leach JL *et al* Nivolumab in patients with metastatic DNA mismatch repair‐deficient or microsatellite instability‐high colorectal cancer (CheckMate 142): An open‐label, multicentre, phase 2 study. Lancet Oncol 2017; 18: 1182–91. 28734759 10.1016/S1470-2045(17)30422-9 PMC6207072

[12] Schrock AB , Ouyang C , Sandhu J *et al* Tumor mutational burden is predictive of response to immune checkpoint inhibitors in MSI‐high metastatic colorectal cancer. Ann Oncol 2019; 30: 1096–1103. 10.1093/annonc/mdz134. 31038663

[13] Ayers M , Lunceford J , Nebozhyn M *et al* IFN‐γ‐related mRNA profile predicts clinical response to PD‐1 blockade. J Clin Invest 2017; 127: 2930–40. 28650338 10.1172/JCI91190 PMC5531419

[14] Tumeh PC , Harview CL , Yearley JH *et al* PD‐1 blockade induces responses by inhibiting adaptive immune resistance. Nature 2014; 515: 568–71. 25428505 10.1038/nature13954 PMC4246418

